# Virtual Reality Training of Social Skills in Adults with Autism Spectrum Disorder: An Examination of Acceptability, Usability, User Experience, Social Skills, and Executive Functions

**DOI:** 10.3390/bs13040336

**Published:** 2023-04-17

**Authors:** Panagiotis Kourtesis, Evangelia-Chrysanthi Kouklari, Petros Roussos, Vasileios Mantas, Katerina Papanikolaou, Christos Skaloumbakas, Artemios Pehlivanidis

**Affiliations:** 1 Department of Psychology, National and Kapodistrian University of Athens, 157 84 Athens, Greece; 2 Department of Psychology, University of Edinburgh, Edinburgh EH8 9AD, UK; 3 Department of Child Psychiatry, Aghia Sophia Children’s Hospital, School of Medicine, National and Kapodistrian University of Athens, 115 27 Athens, Greece; 4 1st Department of Psychiatry, Eginition Hospital, School of Medicine, National and Kapodistrian University of Athens, 115 28 Athens, Greece; 5 Department of Child Psychiatry, P. & A. Kyriakou Children’s Hospital, 115 28 Athens, Greece; 6 Habilis, R&D Team, 141 22 Athens, Greece

**Keywords:** virtual reality, training, autism, social skills, social cognition, executive functions, acceptability, usability, user experience, prompts

## Abstract

Poor social skills in autism spectrum disorder (ASD) are associated with reduced independence in daily life. Current interventions for improving the social skills of individuals with ASD fail to represent the complexity of real-life social settings and situations. Virtual reality (VR) may facilitate social skills training in social environments and situations similar to those in real life; however, more research is needed to elucidate aspects such as the acceptability, usability, and user experience of VR systems in ASD. Twenty-five participants with ASD attended a neuropsychological evaluation and three sessions of VR social skills training, which incorporated five social scenarios with three difficulty levels. Participants reported high acceptability, system usability, and user experience. Significant correlations were observed between performance in social scenarios, self-reports, and executive functions. Working memory and planning ability were significant predictors of the functionality level in ASD and the VR system’s perceived usability, respectively. Yet, performance in social scenarios was the best predictor of usability, acceptability, and functionality level. Planning ability substantially predicted performance in social scenarios, suggesting an implication in social skills. Immersive VR social skills training in individuals with ASD appears to be an appropriate service, but an errorless approach that is adaptive to the individual’s needs should be preferred.

## 1. Introduction

Autism spectrum disorder (ASD) is a lifelong complex neurodevelopmental disorder that significantly impairs individuals’ verbal and nonverbal communication, social interactions, and behaviours (i.e., exhibition of restricted interests, repetitive and unusual sensory–motor behaviours) [1]. Prevalence estimates of ASD have increased over time, and a recent systematic review [2] reported a median global prevalence (ranging within and across regions) of 100/10,000. ASD presents a striking sex difference, as males are more likely to be affected relative to females (3:1 ratio) [3]. The ASD aetiology is suggested to be multifactorial, as both genetic and non-genetic factors (e.g., prenatal/perinatal) may play a crucial role in the manifestation of the disorder (see [4] for a review). In contrast to its first description, ASD is now regarded as a spectrum that spans from very mild to severe [5], as symptoms manifest differently in each individual based on their functionality level (level 1—requiring support; level 2—requiring substantial support; level 3—requiring very substantial support). Nevertheless, several individuals with ASD (not all) require some kind of support throughout their life [5]. Even individuals with high-functioning ASD, similar to other individuals at the mild and lower end of the spectrum, present social skill deficits across the lifespan (up to adulthood). Adults with ASD are likely to experience problems in social and everyday life functioning due to a lack of ecological training and intervention programmes during childhood and adolescence [6].

### 1.1. Social Skills and Executive Functions in ASD 

Adults with ASD have been found to experience social isolation, loneliness, and social anxiety (e.g., [7]) due to their deficient social skills, such as atypical gaze/poor eye contact, less conversational involvement, inappropriate affect, reduced verbal fluency (e.g., [8,9]), poor understanding of social cues, and difficulties in initiating and maintaining social conversation/communication [10]. The social skill deficits in individuals with high-functioning ASD are mainly attributed to impairments in cognitive components such as executive functions (EFs) (e.g., [11]) or cognitive processing speed (e.g., [12]). Indeed, impaired EF, which refers to high-order, goal-directed cognitive processes that control behaviour, thought, and emotions, is another salient characteristic of the spectrum [13]. The EF construct is seen as an umbrella term that includes abilities such as inhibition, working memory, and planning (not an exhaustive list; see [14,15] for a more detailed EF discussion). Two recent meta-analyses [16,17] demonstrated broad EF impairment in ASD, as deficits have been consistently found in several EF aspects (e.g., inhibition, working memory, cognitive flexibility, and planning) across the lifespan.

To implement effective interventions, research over the last decade has aimed to identify which EF aspects contribute to the manifestation of social skills in ASD [11,18], as it is suggested that higher-order cognitive regulation is required for social interactions [19]. EFs have been proposed to support the processing and manipulation of information from one’s and others’ perspectives to facilitate social interaction and communication skills [20]. Such associations are understudied in adulthood in ASD. Limited evidence from childhood and adolescence has shown that performance-based measures of EF (e.g., auditory attention and inhibition/switching) are related to social deficits in ASD (e.g., [21,22]), while ratings-based EFs such as initiation, cognitive flexibility, and working memory were found to be related to adaptive social skills in ASD [23,24]. A recent study [18] also demonstrated significant associations between ratings-based EFs (self-monitoring) and selective social skills (social inferencing and social knowledge) in ASD. It should be noted, though, that none of the aforementioned studies, despite their findings, used in vivo measures of social functioning or a naturalistic context of assessment. Social skills have been theoretically proposed to also depend on social cognition aspects such as mental state/emotion recognition [25], but as these aspects are not consistently associated with social impairment in ASD [26], the extent to which socio-cognitive abilities are associated with the social difficulties in ASD has been debated over the years. Given these potential associations among social cognition and social skills, EFs and social skills, and EFs and social cognition (e.g., [27,28,29]), it has been suggested that EFs may contribute to social skills both directly and indirectly [30]. Social cognition aspects are likely to partially mediate the association between EFs and social skills; perhaps no single cognitive mechanism in ASD can explain the various social difficulties, as previously argued [31], as there may be several factors potentially contributing to social skills (e.g., poor emotion regulation) that could also explain the social and behavioural problems in ASD (e.g., [32]).

### 1.2. Assessment, Training, and Intervention in ASD

The assessment of ASD impairments is critical for identifying potential difficulties and weaknesses when implementing interventions. For example, widely used measures of social functioning include the Social Responsiveness Scale (a measure of general social ability [33,34]), Reading the Mind in the Eyes test (a measure of mental state/emotion recognition [35]), and the Autism Diagnostic Observation Schedule (a measure of social interaction, communication, and play [36,37]). Taking into consideration the tremendous impact of the aforementioned cognitive and social impairments on the everyday lives of individuals with ASD, suitable intervention and training programmes are needed [38]. Targeting cognitive deficits, cognitive training exercises in adults with ASD are usually implemented to enhance performance through repeated practice on EF tasks (e.g., [39,40]). Cognitive training exercises encompass various intervention methods, such as pen-and-paper tasks, downloadable tools, and logical games. Given the EF contribution to several aspects of social functioning, targeting specific EF aspects is thought to improve the effectiveness of training interventions in ASD [41]. However, it should be noted that cognitive training studies in ASD have been designed only in recent years, and thus, their limited and mixed results as well as their lack of ecological validity are the subject of ongoing discussion (e.g., [42,43,44]).

When it comes to social skills, several different strategies have been used in training and intervention programmes to enhance social functioning (usually social interaction and communication) in adults with ASD. For example, strategies such as social stories and social scripts, behavioural modelling and role-playing demonstrations, video modelling, and self-modelling (e.g., [45]) in the context of didactic lessons to enhance conversational skills, developing friendships, the appropriate use of humour, dating, and handling embarrassing feedback and peer pressure (e.g., [46]) have been used in ASD. Most psychosocial intervention and training programmes in ASD, however, are thought to yield limited benefits [47] because of their limited ecological validity, which does not permit the generalisation of the outcomes to everyday life [48,49]. The limitations of the aforementioned methods are thought to likely arise because of the ASD literature’s tendency to examine social (and/or cognitive) deficits as isolated and individual features without evaluating how they manifest in real-life contexts, in which outcomes are influenced by relational dynamics as well [41,50]. For that reason, computing technology with more naturalistic set-ups and role play is a significantly effective training and intervention medium for individuals with ASD [51].

### 1.3. Ecological Validity, Virtual Reality Assessments, and Interventions

Ecological validity refers to the verisimilitude (i.e., the likeness to everyday life) and veridicality (i.e., the association between the observed and real-life performance) of a neuropsychological tool, which subsequently allows the generalisation to everyday life [52]. In contrast to paper-and-pencil or computerised approaches, which incorporate static and simplistic testing and training environments and stimuli, immersive virtual reality (VR) facilitates the attainment of enhanced ecological validity and pleasantness [53]. Immersive VR neuropsychological tools may thus contribute to the understanding of everyday functionality (e.g., [54,55]) and improve everyday physical and cognitive functioning (e.g., [56,57,58]). In the context of VR interventions in ASD, immersive VR technology facilitates the creation of simulated environments that can be used to help individuals with ASD improve social skills, communication, and behaviour [59,60,61,62]. These interventions aim to provide individuals with ASD with a safe and controlled environment in which to practice and develop skills, as well as to reduce the anxiety and stress associated with real-world interactions [60]. VR interventions can include activities such as role-playing social scenarios, virtual social skills training, and virtual exposure therapy. However, the effective implementation of immersive VR for research and clinical purposes requires technological competence [63]. An inappropriate conceptualisation of VR training may have negative ramifications and compromise its otherwise beneficial outcomes [62]. 

Nevertheless, several VR applications have efficaciously been implemented for assessment and intervention purposes. The VR Everyday Assessment Lab assesses everyday memory (prospective and episodic), attention (visuospatial and auditory), and EFs (planning and multitasking) and has been found to be a valid and substantially more pleasant testing experience [53] that is representative of the everyday functionality of adults [54,55]. The ClinicaVR: Classroom-CPT is a VR classroom that examines selective and sustained attention and inhibition, and it has been validated in children and adolescents [64]. Regarding interventions in ASD, there is preliminary evidence postulating its feasibility for being adopted in clinical and educational environments [59,65]. Additionally, the use of social stories in VR has been evaluated by clinicians for implementation in clinical and educational settings for social skills training in children with ASD [66]. Preliminary evidence suggests that VR software may improve the conversational [61], problem-solving, and communication skills of children with ASD [67]. After a VR training protocol, children with ASD showed significant improvements in emotion expression and regulation and socioemotional reciprocity [68]. Comparably, two more studies [69,70] reported a substantial enhancement of social skills in children with ASD after they attended VR-based training sessions. It is important, however, to underline that VR interventions in ASD are still considered an emerging field, and more research is needed to fully understand their efficacy, usability, and the provided user experience, as well as their acceptability by individuals with ASD [59,60,62]. Furthermore, the relationship between performance in VR social scenarios and cognitive functioning has not yet been investigated. Finally, while there are several VR applications used in children and/or adolescents with ASD, none of the aforementioned VR applications was designed for or implemented in adults with ASD. 

### 1.4. VRESS

The VR Enhancement of Social Skills (VRESS) was developed in line with the guidelines for developing VR software for research and clinical applications in the field of psychology [71]; these guidelines have been found to produce VR software that meets the criteria of the American Academy of Clinical Neuropsychology (AACN) and the National Academy of Neuropsychology (NAN) [72]. VRESS incorporates social scenarios that are exemplary of adult activities and common in daily life, such as renewing one’s subscription to the gym, selecting a movie and buying a ticket at the cinema, browsing the available options and purchasing a smartphone at the phone store, attending a seminar class and interacting with the instructor and the co-students, and attending a job interview and responding to the interviewers’ questions. The social scenarios were designed in line with the guidelines of Gray and Garand [73] for providing social stories that provide individuals with ASD (i.e., the learners) a visual representation and a description of a situation or activity to prepare and instruct them on what to expect, as well as the underlying reasons for this matter. Thus, the social scenarios of VRESS are descriptive rather than directive. The social stories were designed for individuals with ASD to comprehend and apply the intricacies of interpersonal communication to interact more appropriately and effectively. The social story approach provides the opportunity for people with ASD to identify the context, discuss their motives, comprehend the amplifiers or the obstacles, and improve their social skills [73,74]. 

### 1.5. Research Aims

For the sake of clarity, we provide a description of the terminology that pertains to the research aims:

- Usability: the capacity of a system to provide a condition for its users to perform the tasks safely, effectively, and efficiently while enjoying the experience.
- User experience: how a user interacts with and experiences a product, system, or service.
- Acceptability: the quality of being satisfactory and able to be agreed to or approved of being software for a specific purpose.

This study thus aimed to:

(1) Evaluate the usability and user experience of an immersive VR training software for social skills (i.e., VRESS) in adults with ASD.
(2) Examine the acceptability of the VR training software of social skills as a social service (i.e., from a service user’s point of view) that may be prescribed and/or offered by clinicians, educators, and social workers to adults with ASD for training and improving their everyday social skills.
(3) Investigate the relationships between cognitive functioning (i.e., aspects of social cognition and EFs), the independence/functionality level of individuals with ASD, performance in VR social scenarios, and acceptability, usability, and user experience ratings.

## 2. Materials and Methods

### 2.1. VRESS Scenarios and Interface

The VRESS software runs on SteamVR and is compatible with every VR headset that runs on the SteamVR platform (e.g., HTC Vive series, HTC Vive Pro series, Oculus Rift series, and Varjo VR series; see here (https://www.businessinsider.com/guides/tech/which-vr-headsets-work-with-steam) for an exhaustive list; accessed on 14 March 2023). VRESS encompasses five social scenarios: (1) being at the gym; (2) buying a smartphone at the phone store; (3) going to the cinema to watch a movie; (4) attending a seminar class; (5) attending a job interview. Each scenario has three different difficulty levels: (1) easy; (2) moderate; and (3) difficult. Thus, the five scenarios have three diverse versions (i.e., per difficulty level), which means that there are a total of fifteen diverse scenarios in VRESS. The difficulty level is determined by the complexity of the scenario in terms of how many social tasks the users have to perform and how many 3D characters they need to interact with (e.g., just buying a ticket or having a discussion with friends about which film they should watch and then buying tickets for everyone). Furthermore, given that visual sensitivity (e.g., to intense light) [75] and agoraphobia and/or social phobia [76] symptoms are highly prevalent in ASD, the difficulty may further be modulated by defining the intensity of lights and the density of the population of 3D Non-Player Characters (NPCs; i.e., 3D characters that the user does not interact with) in the virtual environment. VRESS provides a distinct User Interface (UI) to the operator’s (e.g., clinician, researcher, social worker, or educator) laptop/PC, which is not visible to the immersed user. Thus, beyond rendering the virtual environment that the user is immersed in, VRESS provide a UI to the operator, which allows them to control the VR experience. 

**Figure 1:**
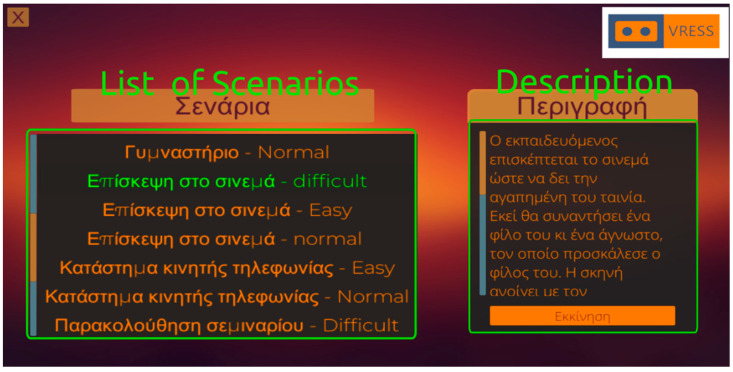
Central User Interface for selecting the social scenario (**Left**) and describing the requirements to the user (**Right**).

**Figure 2:**
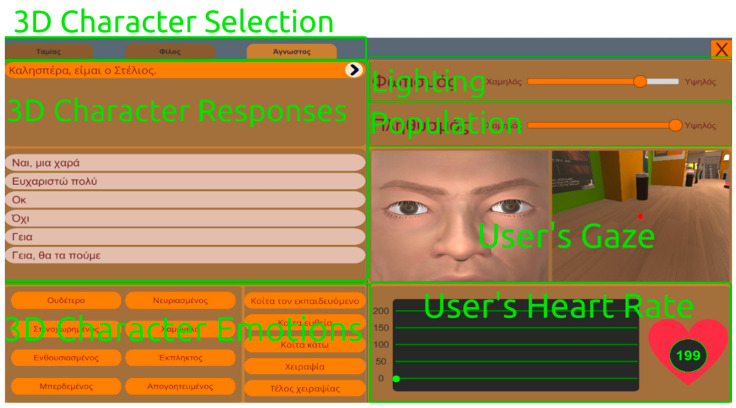
User Interface during the scenario for selecting the interacting 3D character (**Top Left**) and controlling the interacting 3D character’s responses (**Middle Left**) and emotions (**Bottom Left**) and the lighting intensity and NPC population density (**Top Right**) in the virtual environment, as well as for observing the user’s gaze (**Middle Right**) and heart rate (**Bottom Right**).

There are two types of UIs. There is a central UI (see Figure 1), which appears when the VRESS application starts, that provides the operator with the available scenarios and their difficulty levels, as well as the description of each scenario level that has to be given to the users/trainees (i.e., the individuals with ASD) for understanding the social situation and the social tasks that they need to perform. This central UI also appears when the user/trainee completes a social scenario; thus, the operator needs to select and commence the next social scenario that the user/trainee has to perform. While the user/trainee is immersed in a scenario, another UI appears on the operator’s screen (see Figure 2). This UI enables the operator to control which 3D character the user/trainee should interact with (note that there are 1–4 interactable 3D characters per scenario, while the other characters are just bystanders). Additionally, it allows the operator to manage how the interacting 3D character will respond by opting for one of the available responses. Furthermore, the operator may control the 3D character’s facial expressions that correspond to diverse emotional states (e.g., neutral, angry, enthusiastic, sad, happy, confused, disappointed, or surprised), as well as define the gaze direction of the 3D character (e.g., looking at the trainee, straight ahead, or down). As mentioned above, using this UI, the operator may also control the intensity of the lighting and the density of the NPC population (i.e., how many bystanders will populate the virtual environment) in the virtual environment. Finally, given that social anxiety is associated with an increased heart rate [77] and atypical eye contact [78], which are common in ASD, this UI permits the operator to monitor the user’s/trainee’s gaze (i.e., where the trainee is looking, e.g., at the 3D character’s torso or eyes, mouth, and nose) and heart rate. 

#### 2.1.1. Gym

**Figure 3:**
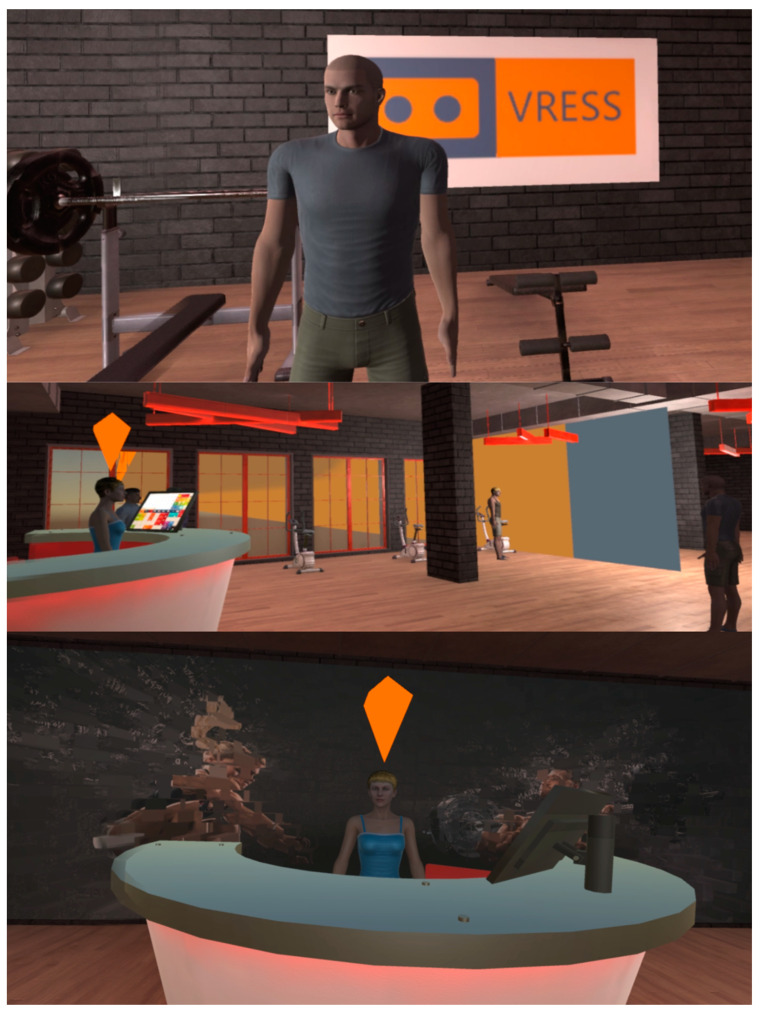
The instructor (**Top**), main area (**Middle**), and reception desk (**Bottom**) of the gym.

In this scenario, the trainee is at the gym (see Figure 3). In the easy mode, the trainee has to ask the gym instructor how to operate the running treadmill. At the moderate level, the trainee has to ask another person (i.e., a co-athlete) at the gym and then the gym instructor how to operate the running treadmill. Finally, at the difficult level, in addition to asking how to operate the running treadmill, the trainee has to renew their subscription to the gym and bargain over the increased fee. 

#### 2.1.2. Phone Store

**Figure 4:**
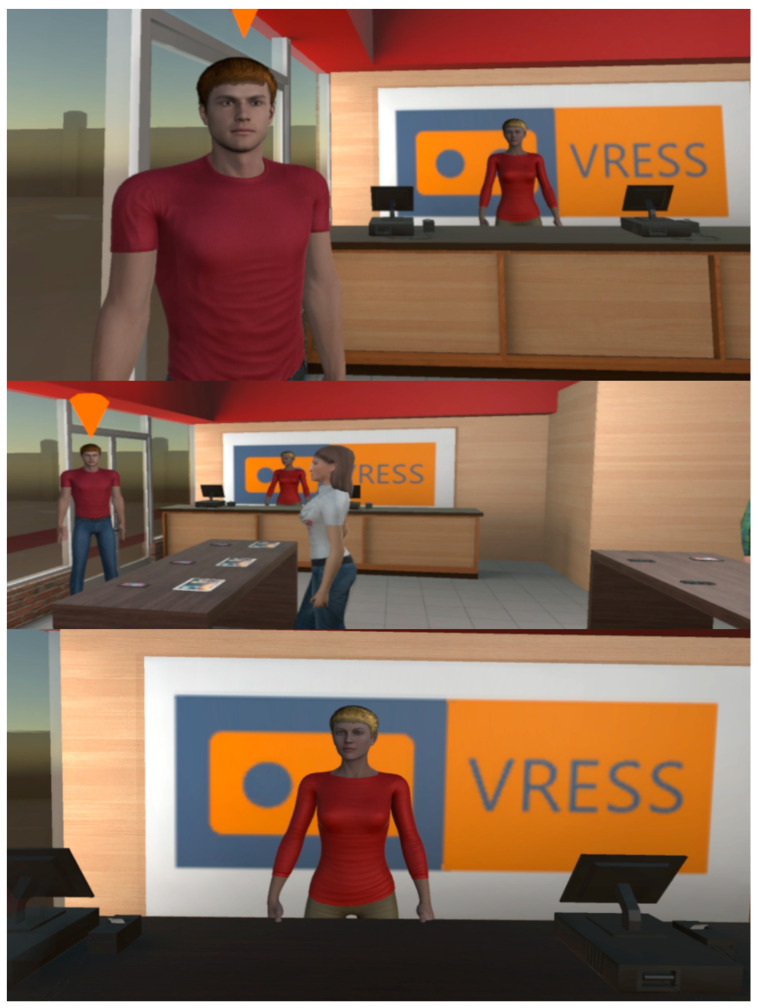
Customer service (**Top**), main area (**Middle**), and cashier (**Bottom**) of the store.

At the phone store (see Figure 4), the trainee has to buy a smartphone that costs up to EUR 200. At the easy level, the examinee just has to browse the available options offered by the customer service person. At the moderate level, while the trainee is instructed that they should buy a specific model of a brand, they have to be open to a special offer for a smartphone with better technological specifications and a lower price. At the hard level, the trainee has to browse all the available options and bargain based on a lower price that they found online.

#### 2.1.3. Cinema

**Figure 5:**
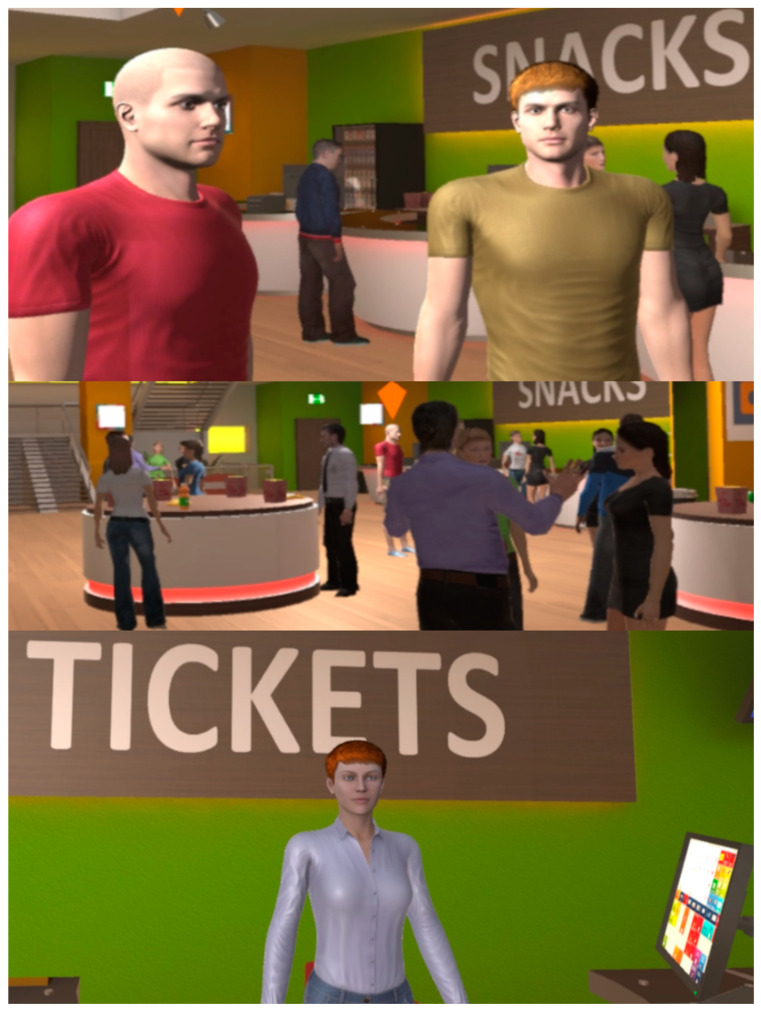
Friends (**Top**), main area (**Middle**), and ticket desk (**Bottom**) of the cinema.

At the cinema (see Figure 5), the trainee has to select a movie and buy a ticket for this movie. At the easy level, while having a specific movie in mind, the trainee arrives late at the cinema, and they need to browse their options (e.g., the next showing or another movie) and buy a ticket. At the moderate level, while having an appointment with a friend, the examinee arrives late, and they need to apologise and then buy tickets for the movie. At the difficult level, while having an appointment with a friend and another person (a friend of the friend), they need to meet them, introduce themselves, discuss finding the way to the cinema and film genres that they like, then choose a movie, and finally buy tickets for everybody. 

#### 2.1.4. Classroom

**Figure 6:**
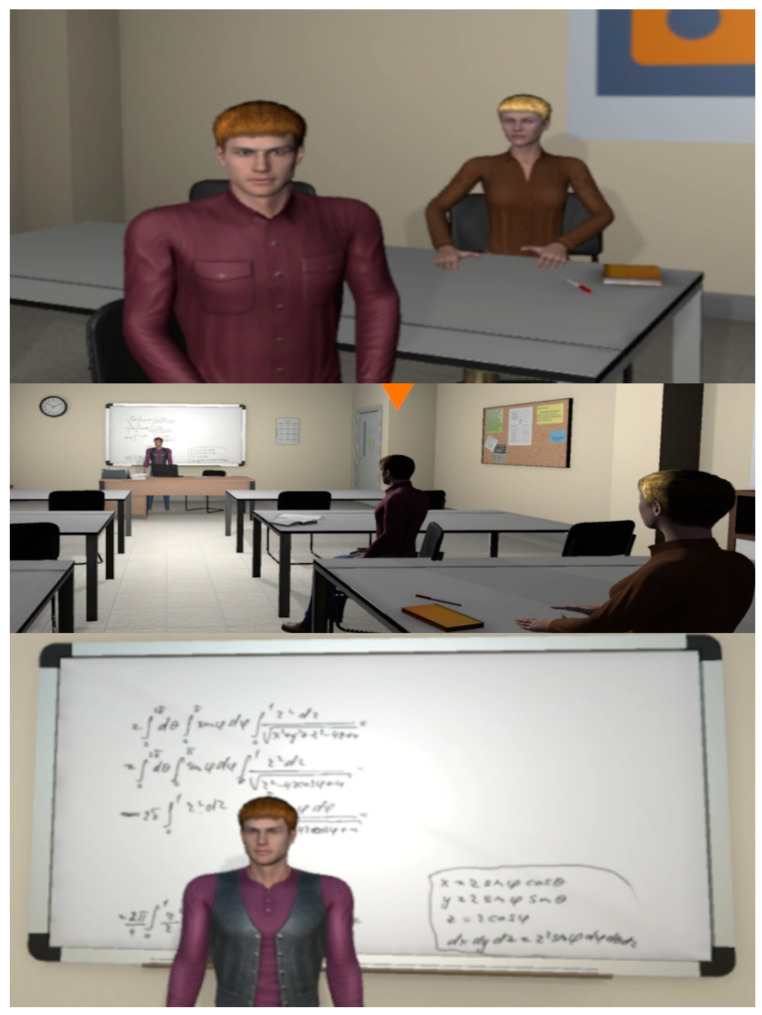
Co-students (**Top**), main area (**Middle**), and lecturer (**Bottom**) of the classroom.

In this scenario, the trainee has to attend a seminar class (see Figure 6). At the easy level, the trainee has to attend a 3 min lecture by the instructor on how to find reliable information on the internet. The trainee has to respond to the instructor’s question, where they have to share their opinion on Wikipedia. At the moderate level, in addition to the aforementioned interaction, the trainee has to interact with their co-students during the break and ask them about their presentation. At the difficult level, the trainee also has to apologise to a co-student for making a mistake, which may undermine the reliability of their co-project and presentation. 

#### 2.1.5. Interview

**Figure 7:**
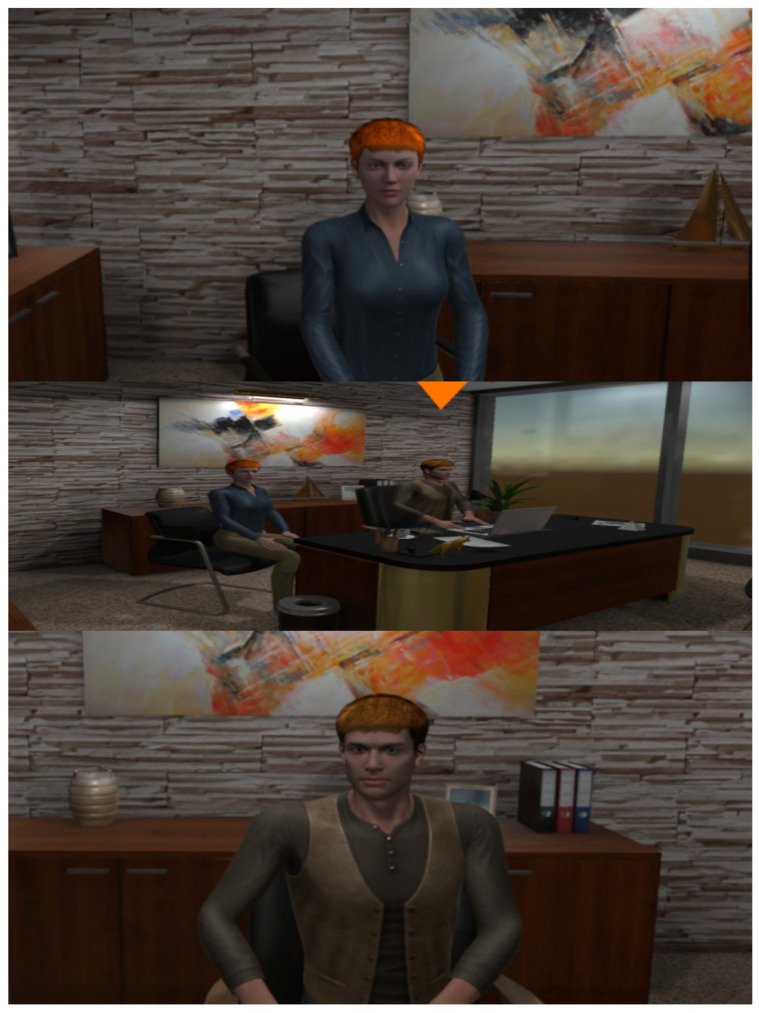
The HR manager (**Top**), main area (**Middle**), and team leader (**Bottom**) of the office.

In this scenario, the trainee has to attend a job interview at the offices of an IT company (see Figure 7). At the easy level, the trainee is required to convince the team leader to hire them as an IT assistant. At the moderate level, the trainee needs to convince both the team leader and the HR manager to hire them as an IT assistant. Finally, at the difficult level, there is one more person in the waiting room, with whom the trainee has to initiate a discussion and extract information that may assist them with getting the job. Then, the trainee has to use this information to convince both the team leader and the HR manager that they are the best candidate for this position. 

### 2.2. Neuropsychological Assessment

#### 2.2.1. Reading the Mind in the Eyes Test (Adult Version)—Mental State/Emotion Recognition

This test [35] measures participants’ mental state/emotion recognition ability. It includes 36 pictures of the eyes (only) of different people, which participants are asked to look at carefully and then choose which one of the four available options around each picture best describes what that person may be feeling/thinking. Successful performance requires participants to correctly attribute the emotional or mental state of each picture. One point was awarded for each correct answer. Scores range from 0 to 36. Reading the Mind in the Eyes has been used in hundreds of studies to date and has been found to have good test–retest reliability [79,80]. 

#### 2.2.2. Tower of London—Planning

The Tower of London [81] was used to measure participants’ planning skills. This test includes two identical wooden boards, one for the researcher and another for the participant. Each board has three wooden beams on which there are three wooden balls: one green, one red, and one blue. Participants are asked to reproduce a series of patterns using the wooden balls only with a certain number of moves each time. Participants have to complete 12 planning problems in total: two 2-move planning problems; two 3-move planning problems; four 4-move planning problems; and four 5-move planning problems. To complete all planning problems successfully, participants must follow two rules. Firstly, each planning problem must be completed in a specific number of moves, and secondly, participants are allowed to remove only one ball from each beam at a time. The number of planning problems completed successfully (adhering to the rules) was recorded. One point was given for each successful completion, and zero points were given if participants failed. This test has been the most commonly used measure of planning across the lifespan [82] and presents good test–retest reliability [83].

#### 2.2.3. Digit Recall—Verbal Working Memory

For verbal working memory, the forward and backward digit span subtests from WAIS-III [84] were administered. Participants have to recall and repeat sequences of random numbers back to the researcher in the same order (e.g., “*Please listen carefully and then repeat the following sequence of numbers back to me in the exact same order: 67893*”). Each number sequence is read at a rate of one number per second. In the backward digit span subtest, participants have to repeat the sequence of numbers in the reverse order (e.g., “*1236*” will be repeated as “*6321*”). In the case of two successfully repeated trials within each block, the examiner proceeds with the next one. Participants were awarded 1 point for each correct trial. Digit span has been extensively researched and is considered to be a highly reliable and valid measure of working memory [84,85].

#### 2.2.4. Stroop Test—Inhibition

The Stroop test [86] is a widely used measure of word-colour interference with two conditions. In the congruent condition, the colour of the ink and the printed name of the colour are the same (e.g., the colour name “yellow” is printed in yellow ink), whereas, in the incongruent condition, the colour of the ink and the printed colour word do not match. The ability to inhibit the cognitive interference occurring when the processing of a particular characteristic of a stimulus impedes the processing of a simultaneous second feature of the stimulus is known as the Stroop effect. This test assesses participants’ ability to produce a counterintuitive response as they are asked to read the colour of the ink in which different colour words are printed, instead of reading the colour word. The response time (in seconds) was recorded. The Stroop test has been found to present high test–retest reliability [87].

### 2.3. Questionnaires

#### 2.3.1. Demographics and IT Skills

The participants then provided their demographic data (age in years, sex, education in years, VR experience, computing experience, and gaming experience) by responding to a custom questionnaire. VR, computing, and gaming experience were calculated by adding scores from two questions (6-item Likert scale) for each one. The first question was regarding the participants’ ability (e.g., 5—highly skilled) to operate a VR system, a computer, and a game. Comparably, the second question was pertinent to the frequency (e.g., 4—once a week) of operating a VR system, a computer, and a game. This method of providing a composite score of ability and frequency has been seen as an effective approach for evaluating the experience of an individual in using a technological medium [71,88]. 

#### 2.3.2. Service User Technology Acceptability Questionnaire

The Service User Technology Acceptability Questionnaire (SUTAQ) is a valid and reliable tool for evaluating the acceptability of a technological mean in a target population that uses or will use this telehealth/telemedicine service [89]. The survey includes 22 questions, rated on a scale of 1 to 6, indicating the level of agreement with the statements provided. The survey is divided into 5 sections, each containing between 3 and 9 questions. The addition of the subscores then formulates a total score. 

#### 2.3.3. User Experience Questionnaire

The short version of the User Experience Questionnaire (UEQ) is a valid tool for evaluating the subjective opinion of users towards the user experience that a technological product facilitates [90]. The UEQ is made up of 26 items that are organised into 6 categories. Each item includes a pair of terms with opposite meanings (e.g., “efficient” and “inefficient”). Participants rate each item on a 7-point Likert scale, with responses ranging from −3 (completely agree with the negative term) to +3 (completely agree with the positive term). Half of the items begin with the positive term, and the other half begin with the negative term, and they are presented in a randomised order. The addition of all the responses forms a total score, representing the overall user experience. 

#### 2.3.4. System Usability Scale

The System Usability Scale (SUS) is a simple and efficient tool for assessing the usability of a system [91]. It is made up of a 10-question survey that utilises a five-point Likert scale for participant responses, ranging from “Strongly Agree” to “Strongly Disagree”. The responses are combined to create a total score, which reflects the usability of the system [92]. The SUS can be used to evaluate a wide range of products and services, such as hardware, software, mobile devices, websites, and applications [92]. 

#### 2.3.5. Cybersickness in Virtual Reality Questionnaire

The Cybersickness in Virtual Reality Questionnaire (CSQ-VR) is a questionnaire that evaluates the symptoms and severity of cybersickness and has been shown to have strong structural and construct validity [93] and convergent validity against other cybersickness measurements [94]. It assesses different sub-types of cybersickness symptoms, such as nausea, disorientation, and oculomotor difficulties. It consists of 6 questions, which are presented on a 7-point Likert scale, ranging from “1—absent feeling” to “7—extreme feeling”. The CSQ-VR produces a total score, calculated by adding all the responses.

### 2.4. Participants

Twenty-five (25) adults (19 males/6 females) with an official diagnosis of ASD, aged between 19 and 52 years (*M(SD)* = 29.96 (9.77)), were recruited to participate in the present study. Participants were all either high- or moderate-functioning (functionality levels 1 and 2 according to DSM-5; [1]), had fluent phrase speech, normal intelligence, and had an official ASD diagnosis based on DSM-5 criteria [1] by psychiatrists in multidisciplinary teams with extensive clinical and research experience among adults with neurodevelopmental disorders (for further information, see [95,96,97]). Exclusion criteria included the presence of acute psychopathology requiring urgent psychiatric treatment as well as a Full-Scale Intelligence Quotient (FSIQ) below 70. Ethical approval for the study was obtained from the hospital’s ethics board, and all participants provided the researchers with written informed consent. All participants were compensated for their participation in this project.

### 2.5. Procedures

Every participant in this study first attended a neuropsychological session, where their cognitive functioning was assessed. Additionally, three VR sessions were attended by the participants, where they were immersed in and performed the VR social scenarios at each difficulty level. At the end of the last VR session, they responded to the questionnaires.

#### 2.5.1. Neuropsychological Session

Participants were assessed using the mental state/emotion recognition test and EF measures during one appointment (60 min) by a researcher. During the neuropsychological assessment, the mental state/emotion recognition test was administered first, whereas the order of the EF tasks was randomised across participants. Participants’ responses were scored at the end of each session. Breaks were included when necessary.

#### 2.5.2. VR Sessions

**Figure 8:**
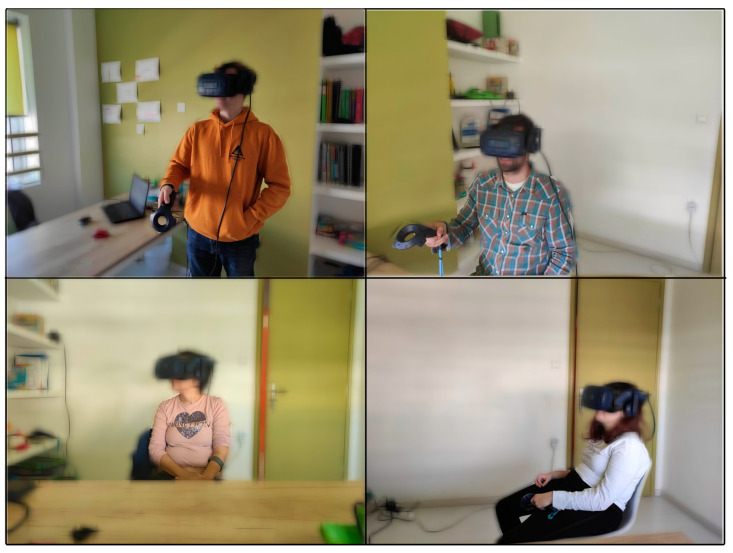
Participants performing the VR social scenarios in a standing or sitting position. Images are blurred to prevent the identification of participants.

Participants were immersed in VRESS by using an HTC Vive Pro Eye headset, which substantially exceeds the recommended hardware criteria for avoiding or alleviating any cybersickness symptomatology [63]. HTC Vive Pro Eye integrates an eye-tracker with a 120 Hz refresh rate and has a tracking accuracy of 0.5°–1.1°. There were three VR sessions per participant, corresponding to the three difficulty levels: easy, moderate, and difficult. Five scenarios were performed in each session. The order of the five scenarios was counterbalanced between the participants (i.e., a complete counterbalance was achieved for every five participants). The order of the scenarios was then the same for the participant in each difficulty-level session. The three different sessions had a week’s gap between them. At the beginning of every VR session, a demonstration of how to properly use and handle the headset and controllers was provided to all participants. The participants performed the social scenarios in a standing or a sitting position (see Figure 8), according to the scenario’s requirement (e.g., classroom and interview required a sitting position). At the end of the third VR session, the participants responded to the questionnaires (see Section 2.3 above), followed by a debriefing session, in which the research aims were explained to them.

#### 2.5.3. Performance Evaluation in the VR Social Scenarios

The researcher who conducted the VR sessions also scored the performance of the participants. As mentioned above (see Section 2.1), the researcher, who was the operator of VRESS, controlled the VR experience. During each scenario, the participants had to interact with the 3D characters in the virtual environment by simply talking to them. The operator of VRESS (i.e., the researcher) then chose the response of the 3D character. The response was the most appropriate to what the participant said to the 3D character. In the case of an inappropriate social interaction by the participant (e.g., saying something irrelevant, being silent, repeating the same thing, or making a faux pas), the operator provided a prompt to the participant to assist them with reacting appropriately. If the participant again did not behave consistently with the social situation’s demands, then the operator opted for a response for the 3D character that would continue the social scenario’s storyline. The performance of the participants was evaluated by two overall scores, the *task completion score* and the *prompts’ score*. The task completion score was calculated as the number of social tasks/interactions that were correctly performed in each social scenario. The participants received 2 points when they appropriately performed all the social tasks/interactions, 1 point when they correctly performed half or more than half of the social interactions, and 0 points when they appropriately performed less than half of the social interactions. A total score for task completion was calculated per difficulty level (i.e., the addition of all points accumulated in the five social scenarios at this difficulty level). An overall task completion score was formed by adding the total scores per difficulty level. Similarly, a prompts’ score was calculated. The number of prompts that were given to the participants in each social scenario was noted. The addition of all prompts per difficulty level formulated a corresponding total score for each difficulty level. The overall prompts’ score was formulated by the sum of all total scores at each difficulty level. 

### 2.6. Statistical Analyses

Descriptive statistical analysis was performed to provide an overview of the sample. Pearson’s correlational analyses were performed to investigate the relationships between cognitive functions, performance in VR social scenarios, and acceptability, usability, and user experience ratings. Kendal’s Tau correlational analyses were conducted to test the associations with the functionality level of individuals with ASD (dichotomous variable, i.e., 1—high-functioning; 2—moderate functioning). Generalised regression analyses were performed to test the ability of the performance variables to predict the functionality level of individuals with ASD. Linear regression analyses were used to examine the predictors of acceptability, usability, user experience, and the number of prompts. The *R* language [98] in R Studio [99] was used to perform the analyses. The *best-Normalize* R package [100] was used to transform and centralise the data since the continuous variables violated the normality assumption. The distribution of the continuous data was then normal. For performing the respective analyses, the *psych* (correlational analyses) [101], *ggplot2* (plots) [102], and *stats* (regression analyses) [98] R packages were used.

## 3. Results

### 3.1. Descriptive Statistics

#### 3.1.1. Demographic Information

The descriptive statistics of the population are displayed in Table 1. The age of participants seems to extend to the whole spectrum of early adulthood (i.e., 20–39 years) and the early half of middle adulthood (i.e., 40–59 years), although the population is predominantly representative of the former. The education level of the participants indicates that the majority had a university (undergraduate), college, or professional post-high school education. Furthermore, the participants experienced no to very mild cybersickness symptoms, suggesting that cybersickness did not interfere with performance or user experience metrics. The VR experience of the population was relatively low. However, the computing experience appears to be on the upper tier of the possible scores, indicating that the participants were experienced in using computers in their daily life. Yet, the gaming experience was balanced, suggesting that the sample consisted equally of both gamers and non-gamers. 

#### 3.1.2. Performance on Neuropsychological Tests and in Social Scenarios

Regarding the performance on the social scenarios of VRESS, the descriptive statistics for the task completion score indicate a ceiling effect (i.e., the vast majority of participants received a high score that is close to or exactly the maximum possible score) and limited variance. On the other hand, the number of prompts required to efficaciously perform the social interactions in every scenario appears to have a greater range and variance, suggesting that it can be a better discriminator of the performance differences among participants. Finally, regarding the performance on neuropsychological tests, the descriptive statistics indicate an intermediate (e.g., digit span backward) or upper intermediate performance on emotional recognition and EF tests. However, the correct responses on the Stroop test reveal a ceiling effect, while the participants’ response times show a greater variance and range on this test. 

#### 3.1.3. Acceptability, User Experience, and Usability Ratings

**Figure 9:**
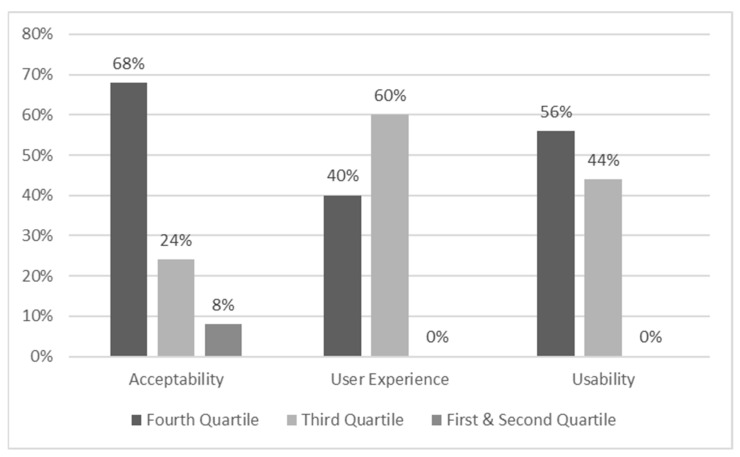
Percentages of the responses per score quartile; quartiles were defined by the maximum possible score of each questionnaire divided by four. Fourth quartile = highest possible scores; first quartile = lowest possible scores.

As Table 1 and Figure 9 illustrate, the vast majority of participants reported very high acceptability of using immersive VR training as a social/health service. A total of 68% of the responses were in the highest quartile, indicating substantially high acceptability. Notably, 92% of the participants’ responses had an overall score above the medium scores of SUTAQ (i.e., 66), which indicates a very high rate of acceptability [103]. For user experience, the majority of responses were in the third quartile (see Figure 9), while all the responses were above the medium score. These scores indicate a high to very high user experience [90,104]. Comparably, 100% of the respondents gave scores in the third and fourth quartiles of the possible scores (see Figure 9), which indicates good to excellent usability [92]. Likewise, both the mean and standard deviation of the usability scores (see Table 1) of VRESS indicate a very good to excellent usability rating [92]. 

### 3.2. Pearson’s and Kendall’s Tau Correlations

#### 3.2.1. Demographics Correlations with Self-Reports and Performance 

The demographic information of participants showed no significant associations with the acceptability, usability, or user experience ratings (see Table 2); however, significant correlations were observed with the performance in social scenarios and neuropsychological tests (see Table 3). Specifically, the participant’s age was positively correlated with the correct responses in the Stroop test, but no other correlations were detected. Similarly, the educational level of the participants revealed positive associations only with the digit span scores and forward and backward recall. Participants’ experience in using VR systems showed no significant correlations with any of the performance metrics. However, both computing and gaming experience were substantially correlated with the performance on RTMIE, suggesting that individuals with more experience using computers and/or playing video games are better at recognising the emotional/mental states of others. In line with this finding, computing experience was also associated with the overall task completion score in VR social scenarios. Finally, experience playing video games was negatively associated with the response time in the Stroop task. 

#### 3.2.2. Self-Reports, Performance Metrics, and ASD Functionality Level

The functionality level of individuals with ASD revealed significant correlations only with the usability rating, the number of prompts required to perform the social scenarios, the performance on digit span forward recall, and the response time in the Stroop test (see Table 4). Specifically, a higher functionality level was associated with higher ratings of the system’s perceived usability, requiring fewer prompts for performing social tasks, having a greater verbal working memory span, and demonstrating faster inhibition. Moreover, substantial positive associations were detected between acceptability, usability, and user experience (see Table 5), suggesting that higher usability of a VR system facilitates a better user experience and increased acceptability as a digital social/health service.

Furthermore, performance in social scenarios and performance on neuropsychological tests were significantly correlated with self-reports on acceptability, usability, and user experience. Requiring more prompts to perform the social interactions in the scenarios was associated with lower acceptability and the system’s perceived usability, as well as with the completion of fewer social tasks. Similarly, a higher task completion score was correlated with higher perceived usability of the system. Usability also revealed positive correlations with both digit span scores (i.e., forward and backward recall; greater working memory span) and Tower of London (i.e., better planning ability) and a negative correlation with the response time in the Stroop test (i.e., faster inhibition). Finally, the number of prompts required to perform the social tasks showed substantial negative associations with digit span forward recall and the Tower of London, suggesting that greater working memory and planning ability each assist in performing social interactions without requiring support and/or reminders. 

### 3.3. Linear Regression and Generalised Linear Models

#### 3.3.1. ASD Functionality Level

**Figure 10:**
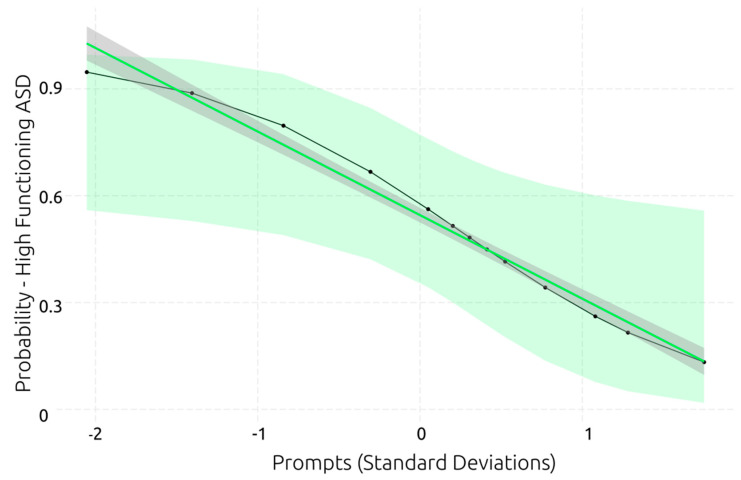
Best generalised linear model for predicting ASD functionality level.

Three models were found to predict the functionality level of individuals with ASD (see Table 6). All models were significantly better than the null model. The models showed high R^2^, indicating that they explain 26–30% of the variance in the functionality level. While all predictors showed large β coefficients, the number of prompts had the highest, suggesting that requiring more prompts substantially predicts a lower functionality level (see Figure 10). Similarly, reduced working memory capacity and slower inhibition each predict a lower functionality level in ASD. 

#### 3.3.2. Performance in VR Social Scenarios 

**Figure 11:**
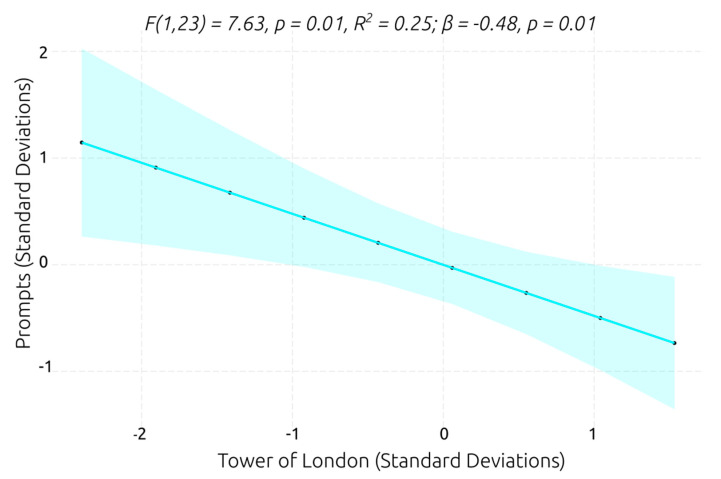
Best linear regression model for predicting prompt number.

Considering that task completion showed a ceiling effect and smaller ranges of scores and variance (see Section 3.1.2), while the number of prompts did not suffer from a ceiling effect and had a large range and rich variance in scores, the number of prompts was preferred as an indicator of performance in VR social scenarios. Only the digit span forward and the Tower of London were significant predictors of the number of prompts. The model with the digit span forward as a predictor was significantly better than the null model, explained 20% of the variance in the number of prompts, and had a relatively large β coefficient (*F*(1,23) = 5.91, *p* = 0.02, *R*^2^ = 0.20; *β* = −0.46, *p* = 0.02), suggesting that a lower verbal working memory span predicts that a greater number of prompts will be required to successfully perform the social tasks in the VR scenarios. However, the score on the Tower of London was the best predictor of the number of prompts (see Figure 11). The model showed that the planning ability explained 25% of the variance in the number of prompts and had a slightly larger β coefficient, which indicates that higher planning ability predicts that a smaller number of prompts will be required to effectively interact and complete the VR social scenarios. 

#### 3.3.3. Service User’s Acceptability and User Experience Ratings

**Figure 12:**
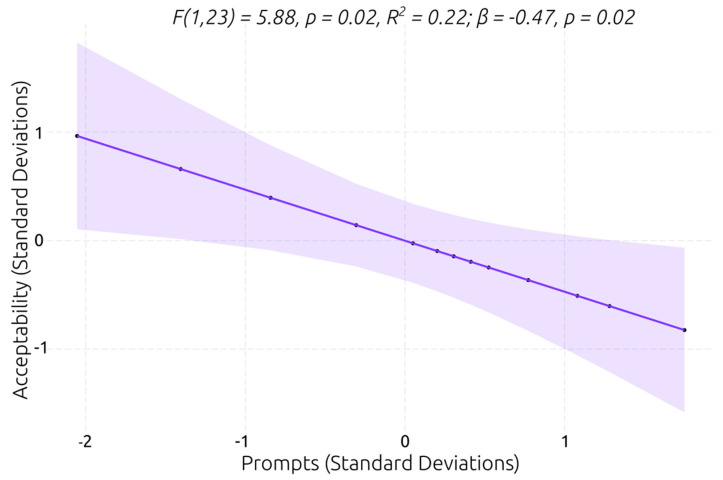
Best linear regression model for predicting service user’s acceptability rating.

**Figure 13:**
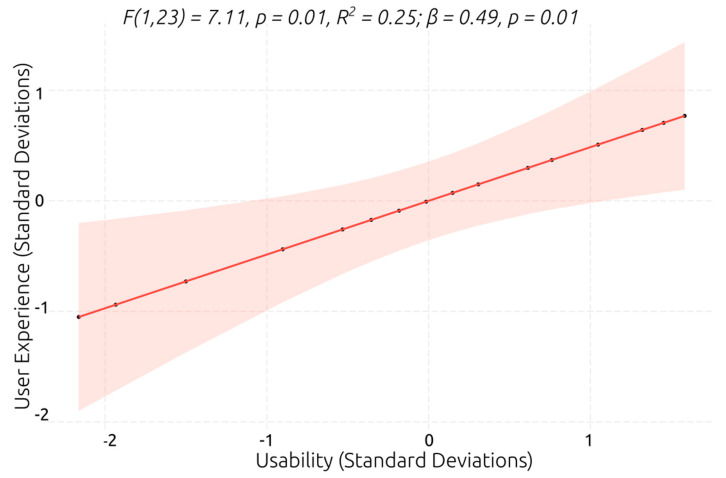
Best linear regression model for predicting user’s experience.

The model with the number of prompts as a predictor of the service users’ acceptability ratings was the only one that was significantly better than the null model (see Figure 12). The model showed that the number of prompts explained 22% of the variance in acceptability ratings and had a relatively large β coefficient, which indicates that individuals with ASD who required more prompts to perform the social scenarios provided lower acceptability ratings. Comparably, the only model for predicting user experience ratings that was substantially better than the null model was the one with the system’s perceived usability rating as a predictor (see Figure 13). The model explained 25% of the variance in the user’s experience rating and had a large β coefficient, suggesting that the individuals who perceived the VR system as having higher usability reported a better user experience. 

#### 3.3.4. System’s Perceived Usability

**Figure 14:**
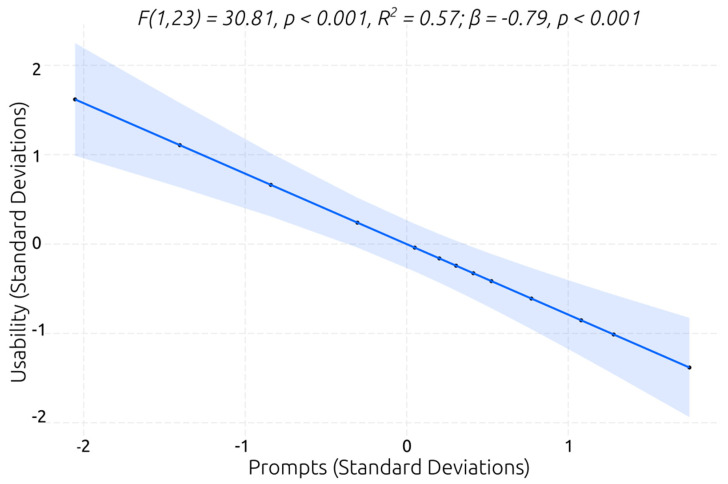
Best linear regression model for predicting system’s perceived usability.

For predicting the system’s perceived usability, four models with a single predictor were identified that were significantly better than the null model (see Table 7). All predictors had large (e.g., task completion) to very large (e.g., number of prompts) β coefficients, indicating that individuals with a better working memory capacity, planning ability, and/or performance in VR social scenarios perceived higher system usability. The overall task completion in the VR social scenarios, digit span forward recall (i.e., verbal working memory capacity), and the Tower of London (i.e., planning ability) explained 27%, 39%, and 47% of the variance in the system’s perceived usability ratings, respectively. However, the best model was the one with the number of prompts as a predictor (see Table 7 and Figure 14). The model explained 57% of the variance in the usability ratings, indicating that the individuals with ASD who required fewer prompts to effectively perform the VR social scenarios perceived higher system usability. 

## 4. Discussion

The present study first aimed to assess the usability, user experience, and acceptability of immersive VR social skills training software (i.e., VRESS) in adults with ASD. The results showed that, in terms of the system’s ratings, the VRESS software exhibited a relatively high performance with positive evaluations, as average scores were close to the high end of the possible scores on questionnaires. Secondly, the examination of the associations between mental state/emotion recognition, EFs, the functionality level of individuals with ASD, performance in VR social scenarios, and self-reported ratings revealed several statistically significant relations. Furthermore, the regression models’ (single predictor) analyses revealed significant predictors of several aspects. The performance in VR social scenarios (i.e., the number of prompts required to effectively perform the social tasks) was the best significant predictor of the ASD functionality level, as well as the ratings of the VR system’s perceived usability and VR social skills training acceptability. Inhibition speed (i.e., the response time in the Stroop task) was also a significant predictor of the ASD functionality level. Working memory (i.e., performance on the digit span forward task) was the second-best predictor of the ASD functionality level and a significant predictor of the VR system’s perceived usability. Finally, the planning ability (i.e., performance on the Tower of London test) was the second-best predictor of the VR system’s perceived usability and the best predictor of performance in VR social scenarios. Overall, the results of this study offer interesting insights into the utility and feasibility of VR social skills training in ASD, the possible implication of EFs in social skills, and the importance of social skills in ASD severity. 

### 4.1. VR Training of Social Skills in Adults with ASD

Based on the authors of the SUTAQ, UXQ, and SUS recommendations for interpreting their scores for technological interventions’ acceptability [103], the quality of the user experience facilitated by the software [90,104], and the system’s usability [92], VRESS showed very high acceptability, user experience, and usability, as rated by participants with ASD. High acceptability suggests that this software [103], which also facilitates remote intervention and the training of social skills, will probably be preferred by adults with ASD. Likewise, the very high usability indicates that the VR software requires a small amount of effort from the user/trainee [92]. In VRESS, the user had only to speak to the 3D characters by using the microphone of the headset and navigate the virtual environment by pressing a single large button on the controller (either left or right). Hence, on the trainees’ part, only a single button was required to be used, while the rest were controlled and operated by the researcher (see Section 2.1. for details). Finally, the high user experience indicates that the VR software offered a highly pleasant and immersive experience to the trainee [90,104]. Given that providing a therapeutic process that is perceived as pleasant and positive by the patients enhances their engagement and commitment to therapy, as well as the effect size of the therapeutic outcomes [105], the high user experience of VRESS suggests that it may achieve comparable positive outcomes. 

Furthermore, given that there is a scarcity of robust evidence supporting the feasibility and acceptability of implementing immersive VR interventions in populations with ASD [59,60,62], the results of this study provide substantial evidence that the implementation of immersive VR social skills training in ASD is feasible and acceptable by adults with ASD. However, it should be noted that VRESS was developed in line with the guidelines for developing VR software for psychological sciences [71]; these guidelines lead to VR software that meets the criteria of AACN and NAN [72]. For this reason, beyond the high ratings in terms of acceptability, user experience, and usability, the participants experienced minimal to absent symptoms of cybersickness, which indicates that VRESS is VR software that meets health and safety criteria. Finally, since VRESS was designed specifically for individuals with ASD, the observed high ratings of acceptability, user experience, and usability highlight the necessity of developing VR software that considers the highly prevalent cognitive and behavioural symptoms of ASD. However, a downside was that the usability and acceptability of VRESS were significantly predicted by the performance in social scenarios. This finding indicates that the negative feeling that was experienced when the participants performed negatively influenced them to rate VRESS with lower scores, while the positive feeling of accomplishment led to more positive scores. Both error correction and errorless learning have been seen as effective in ASD [106]; however, the results of this study suggest that an errorless approach in VR social skills training may result in even higher acceptability and perceived usability. Thus, instead of receiving prompts from the operator/researcher of VRESS, which may be perceived as external corrections, the VR system may provide in-game guidance to promote an errorless completion of social tasks while making the trainees feel that they completed them without external assistance (e.g., with the help of the researcher). Thus, an errorless approach should be preferred in a future iteration of VRESS. 

### 4.2. Demographics’ Role in Cognition

#### 4.2.1. Executive Functions

The results showed that verbal working memory was correlated with the participants’ education. The relationship between digit span scores and education is not surprising, considering that the majority of academic tasks involve reading and lectures, which rely heavily on verbal working memory. Working memory plays an important role in educational attainment, as it is consistently found to predict academic success [107,108]. Involved in the maintenance and processing of information [109], working memory is significantly associated with general reading, comprehension, and mathematical abilities [110,111,112]. In terms of inhibition, the Stroop response time was shown to be significantly associated with gaming experience ratings and usability. The findings of faster inhibition related to higher perceived usability scores could suggest that the ability to suppress automatic responses/ignore distractions more quickly allowed participants to better use and interact with the software. The significant association between gaming experience and the inhibition response time is in line with previous evidence showing that video gamers generally demonstrate faster reaction times and fewer errors relative to non-gamers [113]. Players of action video games were also found to have faster visual and auditory information processing; thus, they presented faster response times than non-gamers [114]. Indeed, practising tasks that rely on inhibition and working memory—such as video games—may lead to improved performance on similar tasks [115].

#### 4.2.2. Mental State/Emotion Recognition

Mental state/emotion recognition ability was not found to be significantly related to performance in VR social scenarios but associated only with computing and gaming experience variables. Previous evidence suggests that individuals with ASD present difficulties in recognising mental states/emotions (e.g., [116,117,118]), but there are limited and mixed findings regarding its association with social competence (e.g., [26]). Generally, as already discussed in the Introduction, socio-cognitive abilities (such as the recognition of mental states/emotions) do not present consistent associations with social skills in ASD. Our results show that, in adults with ASD, it is plausible that other cognitive functions (such as EFs) are more strongly implicated in the expression of social skills. Considering, though, that it could be the case that no single cognitive construct can explain all of the variance in social difficulties in ASD, further research is needed to shed more light on this association. Future studies may also take into consideration other emotional and relational factors that could potentially contribute to social skills. For example, individuals with ASD may have difficulty regulating their emotions (emotional regulation) or sharing others’ feelings (e.g., empathy), which can make it challenging for them to respond appropriately to social cues and situations. Accordingly, low self-esteem, negative interpersonal relationships, or even low social motivation may also play a role in shaping the social skills of individuals with or without ASD. Finally, the correlations between mental state/emotion recognition, gaming experience, and computing experience reveal that individuals who had more experience with video games were more able to recognise mental states/emotions in the RTMIE test. Due to their interactive nature, modern video games offer realistic cinematics and compelling avatars with complex facial expressions, which may enhance gamers’ ability to attribute and recognise emotions and mental states in real-life contexts.

### 4.3. Executive Functions and Social Skills

Gollwitzer’s *implementation intention* pertains to the formulation of an effective plan of action, which incorporates the associations between a cue and the intended action (e.g., if I encounter X, then I will do Y) [119]. Correspondingly, planning ability is an executive function that requires thinking about the future and accordingly organising and prioritising future actions to achieve the desired goal(s) [15,120]. In everyday life, planning defines when and where an action will take place and involves updating/prioritising the plan of action based on acquired information (e.g., I received a notification for my overdue subscription to the gym, so I need to renew it this evening) [120]. As a result, impaired planning ability is highly prevalent in clinical populations with reduced everyday functionality [121,122], as well as in ASD [123,124]. In this study, planning ability was measured by the Tower of London test, which requires individuals to generate an explicit plan of action, including all the necessary steps, to achieve their goal [81]. Planning ability was found to be the best predictor of performance in VR social scenario performance (i.e., the number of prompts). Comparable to everyday life, the VR social scenarios required participants to plan/implement strategies for how to move their bodies, modulate the tone of their voices, express their thoughts and perspectives, and decide with which person they should approach and how they should interact with them to achieve the respective social goals (e.g., choosing a film and buying tickets for it). Participants with ASD who presented lower planning abilities experienced more difficulties in performing the required tasks in these social scenarios. On the other hand, participants with ASD who had better planning ability required fewer prompts to perform the social tasks in VRESS, suggesting that their planning ability assisted them in performing social interactions without requiring support. These results and interpretations are in line with the findings of studies in children with foetal alcohol spectrum disorders [121] and 22q11 deletion syndrome [122], where planning ability was a significant predictor of social skills. Note that, comparable to individuals with ASD [123,124], individuals with these syndromes frequently have impaired social skills and planning abilities [121,122]. Taken together with the results of this and previous studies, planning skills are likely to facilitate social interactions, as individuals need to plan and monitor their own and others’ actions to adjust their responses and behaviours. Successful social interactions thus not only require the manipulation of one’s and others’ perspectives or the processing of social cues (i.e., working memory) but also may need planning abilities to make behavioural decisions and develop strategies. It should be noted at this point that social strategies may involve conscious planning, as discussed above, but of course, social behaviours may also manifest unconsciously (particularly in everyday life), as they are often based on previous interactions or emotional experiences.

In line with a review of studies on working memory impairments in ASD, where lower scores in verbal working memory were associated with greater problems in adaptive behaviour [125], in this study, verbal working memory was correlated with performance in VR social scenarios (i.e., the number of prompts). Performance in situations such as the social interactions presented in VRESS scenarios places high demands on processing, which in turn demands increased controlled attentional processing by the executive system of working memory. Participants with ASD who had higher digit span scores required fewer prompts to perform the social tasks in VRESS, suggesting that working memory may facilitate social interactions without individuals needing support and/or reminders. Cognitive structures such as the recognition and understanding of others’ thoughts, beliefs, and mental/emotional states during social interactions place a heavier load on working memory [126], as individuals have to actively maintain and manipulate personal perspectives and new, complex information from external social cues. Accordingly, social interactions could be considered a dual task (i.e., based on one having to balance personal perspectives with those of the people they are interacting with) and, for that reason, require working memory mechanisms [127]. Taking all these together, it is likely that participants with ASD who have lower working memory abilities required more prompts to complete the social scenarios because effective social cognition and social interaction are not possible unless one can effectively maintain and process perspectives, social cues, and communication strategies. Nevertheless, working memory ability was not a significant predictor of performance in VR social scenarios, suggesting that its implication in social skills may be secondary and/or moderating. Indeed, this interpretation of our findings agrees with the findings of a recent study, where a moderating role of working memory between verbal ability and social skills was observed during the early schooling years, during which the acquisition of social skills is crucial [128]. 

### 4.4. Predictors of Functionality Level in ASD

Our results indicated that the ASD functionality level was related to and predicted by inhibition and verbal working memory, supporting previous evidence that has pinpointed a link between EFs and the severity of later features/symptoms in ASD [129]. Generally, impaired EFs have been proposed to underlie the severity of the core symptoms of the spectrum [125,130]. In line with this evidence, our results suggest that executive functions are central to ASD and highlight their importance as a crucial domain for support and training/intervention. It should be noted at this point, though, that less attention has been generally given to the examination of potential cognitive factors that may be crucial for the implementation of timely and effective interventions in ASD. Future longitudinal studies can further clarify whether executive functions have prognostic significance in adults with ASD. 

Most importantly, though, the performance in VR social scenarios (i.e., number of prompts) was found to be the best significant predictor of the ASD functionality level. Impaired social and communication skills are core features of ASD, which is common across the spectrum regardless of the functionality level [131,132,133,134]. Although some of the best predictors of ASD severity/functionality in childhood are the language level [135] or IQ [136], the severity of social and communication skill impairments has been found to be associated with [131,134] or differ across [132,133] the diverse functionality levels within the ASD diagnosis. Observing the performance in VR social scenarios as a significant predictor of the ASD functionality level is thus aligned with the findings of the aforementioned studies. However, it should be noted that the results of this study indicated that social skills were not just a significant predictor but the best predictor of the functionality level in ASD. Given that the participants of this study were diagnosed with either functionality level 1 or 2 (i.e., high and moderate functioning, respectively) based on DSM-5 [1], this outcome suggests that social skills may potentially serve as a central indicating factor of functionality in high- and moderate-functioning adults with ASD. Notably, the social scenarios of VRESS benefit from enhanced ecological validity, which allows the depiction of everyday functionality [52,72]. Thus, this outcome may be also attributed to the enhanced ecological validity of VRESS social scenarios, which encompass the complexity and the demands of social contexts and situations in daily life. 

### 4.5. Limitations and Future Studies

The findings of the present study should be interpreted considering its limitations. The present sample of adults with ASD may not represent the spectrum of the more general population. Participants’ average age was approximately 30 years, being mostly representative of early adulthood (i.e., 20–39 years old). Future studies should thus establish whether these results can be replicated among younger children, adolescents, and/or older adults. Furthermore, the current study was not a randomised controlled trial (RCT) study to effectively examine the efficacy of VRESS in improving the social skills of individuals with ASD. Future studies should hence consider conducting an RCT experimental protocol, including the incorporation of a control group, to scrutinise the efficacy of VR interventions in enhancing the social skills of adults with ASD. Finally, VRESS did not offer an errorless learning approach, which our results showed may be beneficial for adults with ASD. Future iterations of VRESS should facilitate an errorless learning approach to improve its efficacy. Finally, future VR studies are needed to identify more potential prognostic markers of cognitive and social functioning in ASD.

## 5. Conclusions

VRESS appears to be an appropriate VR social skills training system that facilitates high acceptability, usability, and user experience in individuals with ASD, without inducing adverse symptoms. These positive outcomes pertaining to VRESS also support the effectiveness and feasibility of implementing VR social skills training in individuals with ASD. Furthermore, executive functions were found to be implicated in the social skills of adults with ASD. Finally, social skills were seen to be the best indicator of the severity/functionality level of adults with ASD. 

## Figures and Tables

**Table 1:** Descriptive statistics of the sample.

Variables	Mean (SD)	Range	Maximum Score
Sex (Female/Male)	6/19	-	-
ASD Functionality Level (1/2)	14/11	-	-
Age	29.96 (9.76)	19–52	-
Education	15.88 (2.26)	12–20	-
Cybersickness	7.52 (2.04)	6–14	42
VR Experience	3.48 (1.38)	2–6	12
Computing Experience	8.96 (2.38)	3–12	12
Gaming Experience	6.68 (3.13)	2–12	12
Acceptability	104.28 (21.07)	49–127	132
User Experience	126.00 (26.33)	78–180	180
Usability	77.12 (12.08)	54–98	100
Task Completion Score	27.56 (1.82)	24–30	30
Prompts’ Score	11.68 (4.38)	6–20	-
RTMIE	25.72 (5.21)	8–33	36
Digit Span Forward	9.88 (2.33)	4–14	16
Digit Span Backward	7.52 (2.88)	2–13	14
Tower of London	7.88 (2.06)	3–11	12
Stroop—Correct Responses	48.24 (4.01)	30–50	50
Strop—Response Time *	65.44 (24.99)	36–159	-

RTMIE = Reading the Mind in the Eyes; * measured in seconds.

**Table 2:** Pearson’s correlations between demographics and self-reports.

		Age	Education	VR XP	Computing XP	Gaming XP
**Acceptability**	Pearson’s r	0.345	−0.044	0.071	0.213	−0.141
	*p*-value	0.091	0.834	0.736	0.306	0.503
**User Experience**	Pearson’s r	0.351	−0.340	−0.061	0.096	−0.183
	*p*-value	0.085	0.096	0.771	0.647	0.382
**Usability**	Pearson’s r	0.119	0.031	0.269	0.310	0.169
	*p*-value	0.572	0.884	0.193	0.131	0.420

XP = Experience.

**Table 3:** Pearson’s correlations between demographics and performance metrics.

		Age	Education	VR XP	Computing XP	Gaming XP
**RTMIE**	Pearson’s r	0.059	0.372	0.276	0.427 *	0.503 *
	*p*-value	0.780	0.067	0.181	***0.033***	***0.010***
**DS Forward**	Pearson’s r	−0.064	0.412 *	0.281	0.331	0.348
	*p*-value	0.760	***0.040***	0.173	0.106	0.088
**DS Backward**	Pearson’s r	0.152	0.413 *	0.108	0.195	0.237
	*p*-value	0.469	***0.040***	0.607	0.349	0.255
**ToL**	Pearson’s r	0.206	0.349	0.356	0.393	0.349
	*p*-value	0.323	0.088	0.081	0.052	0.087
**Stroop CR**	Pearson’s r	0.411 *	−0.049	0.100	0.267	0.193
	*p*-value	***0.041***	0.815	0.635	0.197	0.354
**Stroop RT**	Pearson’s r	0.037	−0.227	−0.340	−0.380	−0.483 *
	*p*-value	0.860	0.276	0.097	0.061	***0.015***
**Prompts**	Pearson’s r	−0.064	−0.096	0.347	−0.169	−0.115
	*p*-value	0.760	0.647	0.059	0.419	0.585
**Task Completion**	Pearson’s r	0.177	0.206	−0.392	0.468 *	0.196
	*p*-value	0.396	0.324	0.053	***0.018***	0.349

XP = experience; * *p* < 0.05; Significant results are displayed in **Bold** and *Italics*.

**Table 4:** Significant Kendall’s Tau correlations with ASD functionality level.

		Usability	Prompts	DS Forward	Stroop RT
**ASD** **Functionality Level**	Kendall’s Tau B	0.488 **	−0.406 *	0.416 *	−0.365 *
	*p*-value	***0.005***	***0.021***	***0.021***	***0.033***

DS = digit span; RT = response time; * *p* < 0.05, ** *p* < 0.01; Significant results are displayed in **Bold** and *Italics*.

**Table 5:** Pearson’s correlations between self-reports and performance metrics.

		Acceptability	User Experience	Usability	Prompts	Task Completion
**Acceptability**	Pearson’s r	-	-	-	-	-
	*p*-value	-	-	-	-	-
**User Experience**	Pearson’s r	0.534 **	-	-	-	-
	*p*-value	***0.006***	-	-	-	-
**Usability**	Pearson’s r	0.693 ***	0.486 *	-	-	-
	*p*-value	***<0.001***	***0.014***	-	-	-
**Prompts**	Pearson’s r	−0.451 *	−0.200	−0.757 ***	-	-
	*p*-value	***0.024***	0.339	***<0.001***	-	-
**Task Completion**	Pearson’s r	0.366	0.272	0.523 **	−0.635 ***	-
	*p*-value	0.072	0.189	***0.007***	***<0.001***	-
**RTMIE**	Pearson’s r	−0.076	−0.158	0.004	−0.014	0.107
	*p*-value	0.716	0.452	0.987	0.947	0.611
**DS Forward**	Pearson’s r	0.387	0.004	0.628 ***	−0.452 *	0.285
	*p*-value	0.056	0.986	***<0.001***	***0.023***	0.167
**DS Backward**	Pearson’s r	0.228	0.072	0.477 **	−0.299	0.207
	*p*-value	0.273	0.733	***0.016***	0.146	0.321
**ToL**	Pearson’s r	0.354	0.001	0.685 ***	−0.499 *	0.262
	*p*-value	0.083	0.995	***<0.001***	***0.011***	0.206
**Stroop CR**	Pearson’s r	0.039	0.145	0.182	−0.187	0.370
	*p*-value	0.852	0.490	0.383	0.370	0.069
**Stroop RT**	Pearson’s r	−0.203	0.032	−0.569 **	0.313	0.118
	*p*-value	0.330	0.879	***0.003***	0.128	0.576

RTMIE = Reading the Mind in the Eyes test.; DS = digit span; ToL = Tower of London; CR = correct responses; RT = response time; * *p* < 0.05, ** *p* < 0.01, *** *p* < 0.001; Significant results are displayed in **Bold** and *Italics*.

**Table 6:** Best generalised linear models for predicting ASD functionality level.

Predictor	χ^2^	*p*-Value (χ^2^)	β Coefficient	*p*-Value (β)	R^2^
Prompts	6.22	0.01 *	−1.25	0.03 *	0.30
DS Forward	5.83	0.02 *	1.22	0.04 *	0.28
Stroop RT	5.30	0.02 *	−1.09	0.04 *	0.26

DS = digit span; RT = response time; * *p* < 0.05

**Table 7:** Linear regression: best models for predicting system’s perceived usability.

Predictor	F	*p*-Value (F)	β Coefficient	*p*-Value (β)	R^2^
Prompts	30.81	<0.001 ***	−0.79	<0.001 ***	0.57
ToL	20.37	<0.001 ***	0.69	<0.001 ***	0.47
DS Forward	14.98	<0.001 ***	0.67	<0.001 ***	0.39
Task Completion	8.64	0.01 **	0.52	0.01 **	0.27

DS = digit span; ToL = Tower of London; ** *p* < 0.01, *** *p* < 0.001.

## Data Availability

The data presented in this study are available on request from the corresponding author. The data are not publicly available due to ethical approval requirements.
